# Anterior transpetrosal approach for a recurrent craniopharyngioma in a pediatric patient: two-dimensional operative video

**DOI:** 10.3171/2026.7.FOCVID26128

**Published:** 2026-10-01

**Authors:** Florencia Rodriguez Basili, Vera Vigo, Maohua Ding, Masaki Ikegami, H. Westley Phillips, Juan Carlos Fernandez-Miranda

**Affiliations:** 1 Department of Neurosurgery, Stanford University Hospital, Stanford; and; 2 Department of Pediatric Neurosurgery, Stanford University Hospital, Stanford, California

**Keywords:** craniopharyngioma, anterior transpetrosal approach, Kawase approach, petroclival surgery, brainstem surgery

## Abstract

The authors present the case of a 13-year-old patient with a recurrent cystic lesion extending into the lateral pons. A right frontotemporal craniotomy with zygomatic osteotomy and an anterior transpetrosal approach, including extradural middle fossa peeling, Kawase’s triangle petrosectomy, and transtentorial extension, provided a lateral trajectory to the brainstem. Microsurgical dissection allowed safe separation of the lesion from the superior cerebellar artery, trigeminal rootlets, and pontine surface, achieving gross-total resection. This case highlights key technical nuances of anterior transpetrosal surgery for recurrent skull base craniopharyngioma with pontine invasion.

The video can be found here: https://stream.cadmore.media/r10.3171/2026.7.FOCVID26128

## Transcript

Here, we present a case of an anterior transpetrosal approach for a recurrent craniopharyngioma invading the lateral pons in a pediatric patient. This video highlights the surgical rationale, anatomical corridor, skull base exposure, and microsurgical resection.

### 0:37 Clinical Presentation.

The patient was a 13-year-old female who initially presented with headaches, neck pain, shoulder pain, papilledema, and obstructive hydrocephalus. At that time, she was treated through an endoscopic endonasal approach with complete resection of a tuberoinfundibular craniopharyngioma with hypothalamic involvement. Following the initial surgery, she remained without additional neurological deficits except for panhypopituitarism. Pathology of the initial resection confirmed adamantinomatous craniopharyngioma, CNS WHO grade 1.

### 1:11 Surveillance MRI.

Surveillance MRI demonstrated a recurrent cystic lesion located at the right lateral pontine region with extension into the prepontine cistern. The lesion appeared to invade the surface of the brainstem and demonstrated significant mass effect over the pons. Imaging characteristics were consistent with recurrent cystic craniopharyngioma.

### 1:31 Preoperative Neurological Exam.

At the time of recurrence, the patient remained neurologically intact without major focal deficits. Because of the posterior extension into the prepontine region and the prior endonasal surgery, a repeat endoscopic endonasal corridor was considered suboptimal.

### 1:47 Rationale for the Procedure.

A right anterior transpetrosal approach was selected to provide a direct lateral trajectory toward the ventrolateral pons.^1–6^ This approach allows wide exposure of the petroclival region while minimizing temporal lobe retraction and preserving critical neurovascular structures. The primary surgical goals were maximal safe resection, decompression of the brainstem, and preservation of cranial nerve and vascular anatomy.

### 2:12 Anatomy.

Using three-dimensional photogrammetry models, we demonstrate the anterior transpetrosal approach. A right orbitozygomatic craniotomy with extradural middle fossa peeling and anterior petrosectomy through Kawase’s triangle was performed, preserving the greater superficial petrosal nerve and critical skull base anatomy.^7^ Following transtentorial extension, the surgical corridor was developed through a safe entry zone along the anterolateral pontine surface, providing direct access to the tumor-brainstem interface.

### 2:42 Surgical Procedure.

The patient was positioned supine with the head rotated approximately 45° contralaterally. We used a right frontotemporal craniotomy with zygomatic osteotomy. Interfascial dissection allowed preservation of the frontal branch of the facial nerve. An external ventricular drain was placed for brain relaxation. Intraoperative multimodal neurophysiological monitoring was utilized, including motor and somatosensory evoked potentials.

### 3:11 Stepwise Overview.

Before proceeding with the dissection, we summarize the key steps of this approach: extradural middle fossa peeling exposing the greater superficial petrosal nerve, V2, V3, and Meckel’s cave; foramen ovale drilling and trigeminal cistern opening; Doppler localization of the petrous internal carotid artery; anterior petrosectomy through Kawase’s triangle; posterior fossa dural opening; a transtentorial incision; tumor decompression and extracapsular resection; and finally, dural and bony reconstruction.

### 3:44 Surgical Procedure.

Extradural middle fossa peeling, beginning with sectioning of the meningo-orbital band and progressing posteriorly toward the middle meningeal artery, allowed exposure of the greater superficial petrosal nerve, V2, V3, and Meckel’s cave. The foramen ovale was drilled to allow mobilization of V3. Then the trigeminal cistern was opened to release cerebrospinal fluid and further improve temporal lobe relaxation. Doppler ultrasonography was used beneath V3 to localize the petrous internal carotid artery. Extensive anterior petrosectomy was subsequently performed under microscopic visualization until the posterior fossa dura and internal auditory canal dura were fully exposed.

The posterior fossa dura inferior to Kawase’s triangle was opened first, allowing cerebrospinal fluid release and additional brain relaxation. The dura was then opened parallel and superior to the superior petrosal sinus, exposing the basal temporal surface and supratentorial corridor. Subsequently, the dura overlying Meckel’s cave was disconnected to fully communicate the middle and posterior fossa compartments and expose the trigeminal nerve along its cisternal segment.

A transtentorial incision was then performed from anterior to posterior, connecting the supratentorial and infratentorial corridors and significantly expanding the surgical exposure toward the lateral pontine region.^8^

During the final portion of the tentorial cut, a small arterial bleeding arising from the tentorial surface was encountered and successfully controlled using bipolar coagulation without further complication.

This combined transtentorial anterior transpetrosal exposure provided direct visualization of the trigeminal root entry zone, superior cerebellar artery, and the tumor-brainstem interface.

### 5:56 Tumor Resection.

The recurrent cystic craniopharyngioma was identified invading the lateral pontine surface. After decompression of the cystic contents, characteristic "motor oil" fluid was evacuated. Although cyst decompression facilitated surgical exposure, cyst drainage alone is insufficient in recurrent craniopharyngioma. The superior cerebellar artery and trigeminal nerve rootlets were severely adherent to the tumor capsule. Careful microsurgical extracapsular dissection was performed over multiple hours.

Progressive piecemeal capsule resection allowed safe separation from the brainstem. Despite prior surgery and severe arachnoid scarring, all major neurovascular structures were preserved.

The tumor had clearly violated the pial surface and extended into the pontine parenchyma. Meticulous microdissection was required to identify the interface between the tumor capsule and brainstem white matter. All perforating vessels to the brainstem were preserved. The trigeminal nerve fibers and superior cerebellar artery remained anatomically intact throughout the dissection. Continuous neurophysiological monitoring remained stable during the entire procedure.

### 7:24 Reconstruction.

After complete tumor resection and meticulous hemostasis, reconstruction was initiated. Dural reconstruction was performed using layered collagen matrix grafts.

An abdominal fat graft was harvested and placed along the middle fossa floor to reduce the risk of cerebrospinal fluid leakage and pseudomeningocele. The orbitozygomatic bone flap and zygomatic arch were reconstructed using plates and screws.

### 7:39 Postoperative Exam.

Postoperatively, the patient awoke neurologically stable without new focal deficits. Cranial nerve function remained intact, including preservation of trigeminal nerve function despite the extensive dissection along the lateral pontine surface.

Motor and sensory examinations remained unchanged from baseline, and intraoperative neurophysiological monitoring remained stable throughout the procedure. Postoperative MRI demonstrated gross-total resection with complete decompression of the ventrolateral pons and no evidence of hemorrhagic or ischemic complications. Pathology confirmed recurrent adamantinomatous craniopharyngioma, CNS WHO grade 1, consistent with the initial diagnosis.

### 8:20 Conclusions.

This case demonstrates the utility of the anterior transpetrosal approach for recurrent craniopharyngioma involving the ventrolateral brainstem. The combination of extradural middle fossa peeling, anterior petrosectomy, and transtentorial extension provides a direct surgical corridor to the petroclival and lateral pontine regions.

Careful skull base anatomical understanding, extradural petrosectomy, and meticulous microsurgical technique are essential for safe resection of complex recurrent lesions invading the pons.

## Acknowledgments

We used Claude (Claude Sonnet 5, Anthropic) to assist with editing, formatting, and reorganizing the transcript text, time stamps, and reference list in response to reviewer comments. We reviewed, verified, and approved all AI-assisted content. TTSMaker (TTSMaker.com) was used to generate the synthetic audio narration for the video. Dr. Fernandez-Miranda reviewed and approved all narration content for accuracy prior to finalizing the video.

## Disclosures

J. Fernandez-Miranda reported personal fees from Medtronic and KLS Martin, and nonfinancial support from Stryker and Storz outside the submitted work.

## Author Contributions

Primary surgeon: Fernandez-Miranda. Assistant surgeon: Phillips. Editing and drafting the video and abstract: Rodriguez Basili, Vigo, Ding. Critically revising the work: Fernandez-Miranda, Rodriguez Basili, Vigo, Ding, Ikegami. Reviewed submitted version of the work: Fernandez-Miranda, Rodriguez Basili, Ikegami. Approved the final version of the work on behalf of all authors: Fernandez-Miranda. Supervision: Fernandez-Miranda.

## Supplemental Information

### Patient Informed Consent

The necessary patient informed consent was obtained in this study.
